# Repeated hepatic resections and radio-frequency ablations may improve the survival of adult undifferentiated embryonal sarcoma of the liver: report of two cases

**DOI:** 10.1186/s40792-015-0056-y

**Published:** 2015-06-27

**Authors:** Toshiro Masuda, Toru Beppu, Koichi Doi, Tatsunori Miyata, Shigeki Nakagawa, Hirohisa Okabe, Hiromitsu Hayashi, Takatoshi Ishiko, Ken-ichi Iyama, Hideo Baba

**Affiliations:** Department of Gastroenterological Surgery, Graduate School of Medical Sciences, Kumamoto University, 1-1-1, Honjo, Chuo-ku, Kumamoto, 860-0811 Japan; Department of Multidisciplinary Treatment for Gastroenterological Cancer, Kumamoto University Hospital, Kumamoto, Japan; Department of Surgical Pathology, Kumamoto University Hospital, Kumamoto, Japan

**Keywords:** Undifferentiated embryonal sarcoma, Hepatic resection, Radio-frequency ablation

## Abstract

Undifferentiated embryonal sarcoma of the liver (UESL) in adults, especially over 30 years old, is quite rare. We report two adult UESL patients that one of them survived 62 months and one is now surviving more than 65 months treated with repeated hepatic resections and radio-frequency ablations. Although UESL is an entirely unusual and aggressive tumor, multidisciplinary treatments including repeated hepatic resections and radio-frequency ablations may provide a longer survival.

## Background

Undifferentiated embryonal sarcoma of the liver (UESL) is a rare malignant tumor commonly observed in children [1]. UESL in adults, especially more than 30 years old, is entirely uncommon [2, 3]. Because of its infrequency, treatment strategy of UESL in adult is not well established [3, 4]. We have reported short-term outcomes of two adult patients of UESL treated with hepatic resection separately in Japanese literatures [5, 6]. In one of those reports, we emphasized that the patient was successfully treated with a major hepatic resection even for the huge tumor with congestion of three hepatic veins and inferior vena cava [5]. The other reported the pathological feature of the case of UESL [6]. We herein report the long-term outcomes of two cases of adult UESL treated with multiple courses of hepatic resections and radio-frequency ablations (RFAs) for the repeated recurrent diseases.

## Case presentation

### Case 1

**Fig. 1:**
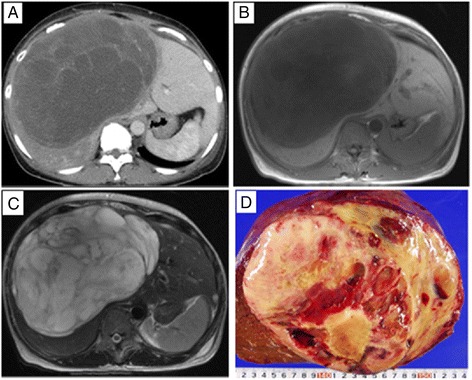
Computed tomography showed a huge low-density tumor sized 17 cm in diameter that showed little enhancement (**a**). Magnetic resonance imaging showed a multicystic tumor to be hypointense on T1-weighted images (**b**) and hyperintense on T2-weighted images (**c**). Resected specimen showed a heterogenic tumor, 23 × 22 × 11 cm in diameter (**d**)

**Fig. 2:**
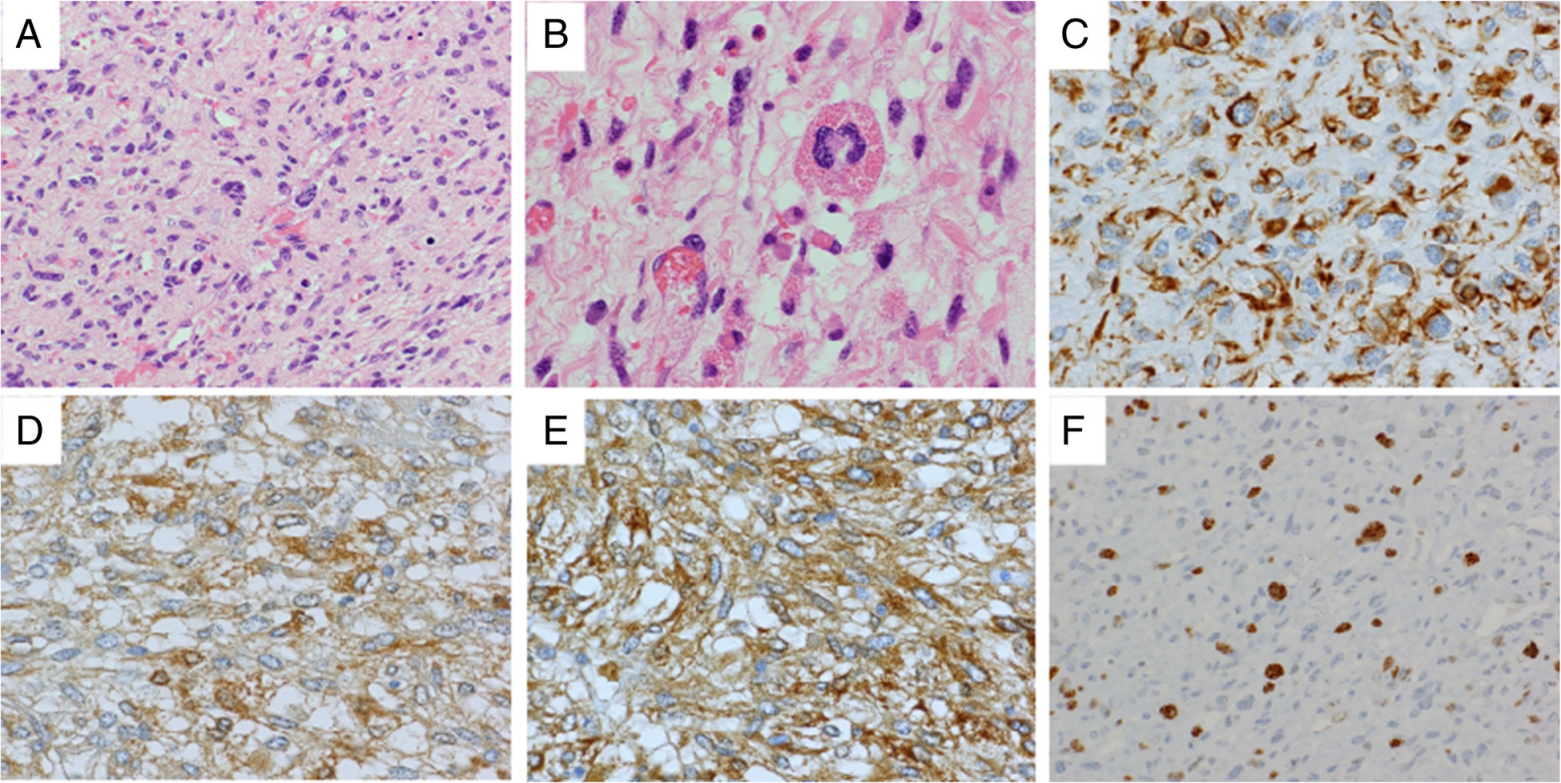
Histological features of HE staining (×200, **a**; ×400, **b**) showed spindled, oval, or stellate tumor cells distributed in myxoid or fibrous stroma. Multiple varying-sized cytoplasmic eosinophilic globules were seen. Tumor cells were positive for vimentin (×400, **c**), alpha 1-antitrypsin (×400, **d**), and alpha 1-antichymotrypsin (×400, **e**). The MIB-1 index was 20 % (×200, **f**)

**Fig. 3:**
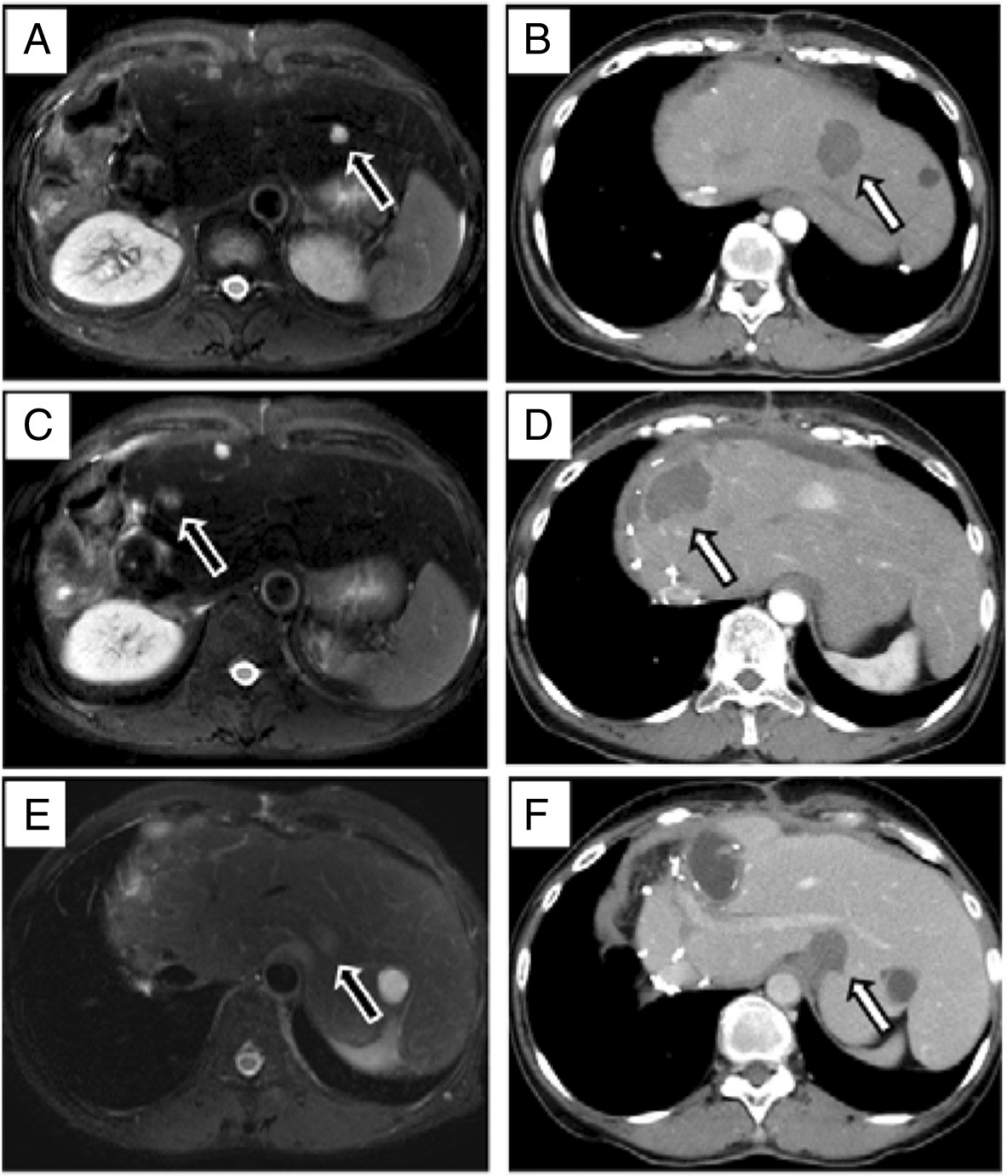
Three recurred tumors (*arrows*; **a, c, e**) were successfully treated with radio-frequency ablations (*arrows*; **b, d, f**), respectively

A 52-year-old female was admitted to the hospital to treat a huge liver tumor with complaints of dyspnea, severe abdominal distension, and lower-extremity edema. Hepatitis B virus surface antigen (HBs-Ag) and hepatitis C virus antibody (HCV-Ab) were negative. The serum level of DUPAN-II was 810 U/ml and that of CA125 was 157 IU/ml, and alpha-fetoprotein (AFP), protein induced by vitamin K absence (PIVKA-II), carcinoembryonic antigen (CEA), and carbohydrate antigen 19–9 (CA19-9) were in the normal range. Computed tomography (CT) showed a huge low-density tumor with a clear margin, 23 cm in diameter, and showed little enhancement after administration of contrast medium (Fig. 1a). Magnetic resonance (MR) imaging demonstrated the multicystic tumor to be hypointense on T1-weighted images (Fig. 1b) and hyperintense on T2-weighted images (Fig. 1c). A right tri-sectionectomy as the first operation was performed safely and successfully, even though the tumor entirely compressed the inferior vena cava and the root of three hepatic veins. Operation time was 13 h and intraoperative bleeding was 2400 g. Resected specimen showed a heterogenic tumor sized 23 × 22 × 11 cm (Fig. 1d). Histologically, spindled, oval, or stellate tumor cells were distributed in myxoid or fibrous stroma. Multiple varying-sized cytoplasmic eosinophilic globules were seen (Fig. 2a, b). Immunohistologically, tumor cells were positive for vimentin (Fig. 2c), alpha 1-antitrypsin (Fig. 2d), and alpha 1-antichymotrypsin (Fig. 2e), and partially positive for alpha-smooth muscle actin (SMA) and CD68/kp-1. S-100, calponin, cytokeratin, factor VIII, CD34, and AFP were negative in tumor cells. The MIB-1 index was 20 % (Fig. 2f). Finally, the tumor was histologically diagnosed as a UESL, and the liver parenchyma was normal liver. Twelve and 19 months after the first operation, for the sequential recurrent diseases, a left caudate lobectomy and a partial hepatectomy of segment 3 were performed as the second and the third surgery, respectively. Twenty months after the first operation, twice transarterial chemoembolizations (TACEs) with CDDP 50 mg + lipiodol 2.5 ml, epirubicin 30 mg, and gelatin sponge were performed. Twenty-five months after the first operation, a radio-frequency ablation (RFA) was performed (pre, Fig. 3a, c; post, Fig. 3b, d). Thirty-five, 42, and 47 months after the first operation, two times of partial hepatic resections and a hepatectomy in combination with RFA (pre, Fig. 3e; post, Fig. 3f) were additionally performed. Because of bone metastases and tumor thrombosis in azygos vein, she unfortunately died 62 months after the initial hepatectomy.

### Case 2

**Fig. 4:**
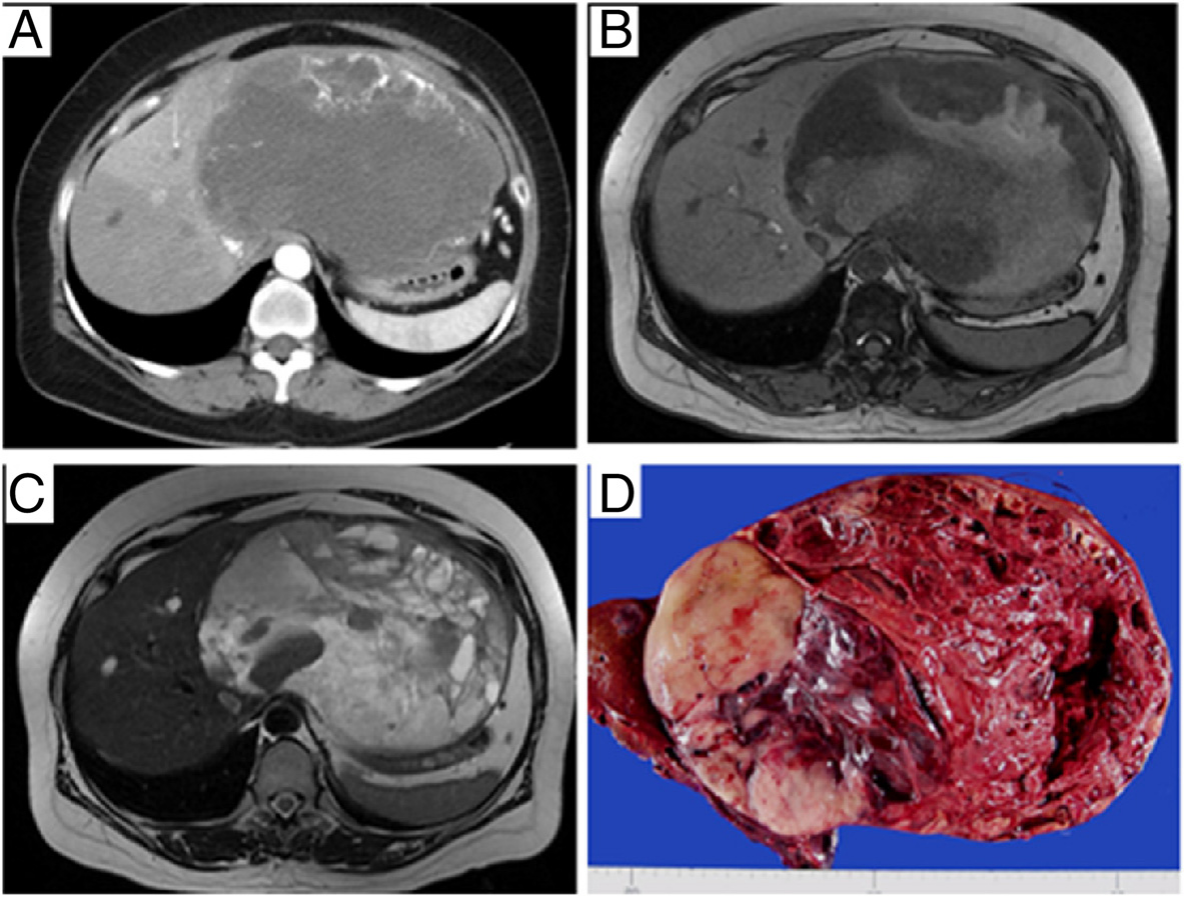
Computed tomography showed a huge low-density tumor sized 18 × 14 cm that showed mild enhancement (**a**). Magnetic resonance imaging showed the tumor to be hypointense on T1-weighted images (**b**) and mixed hyperintense on T2-weighted images (**c**). Resected specimen revealed a tumor with multiple components sized 22 × 19 × 14 cm (**d**)

**Fig. 5:**
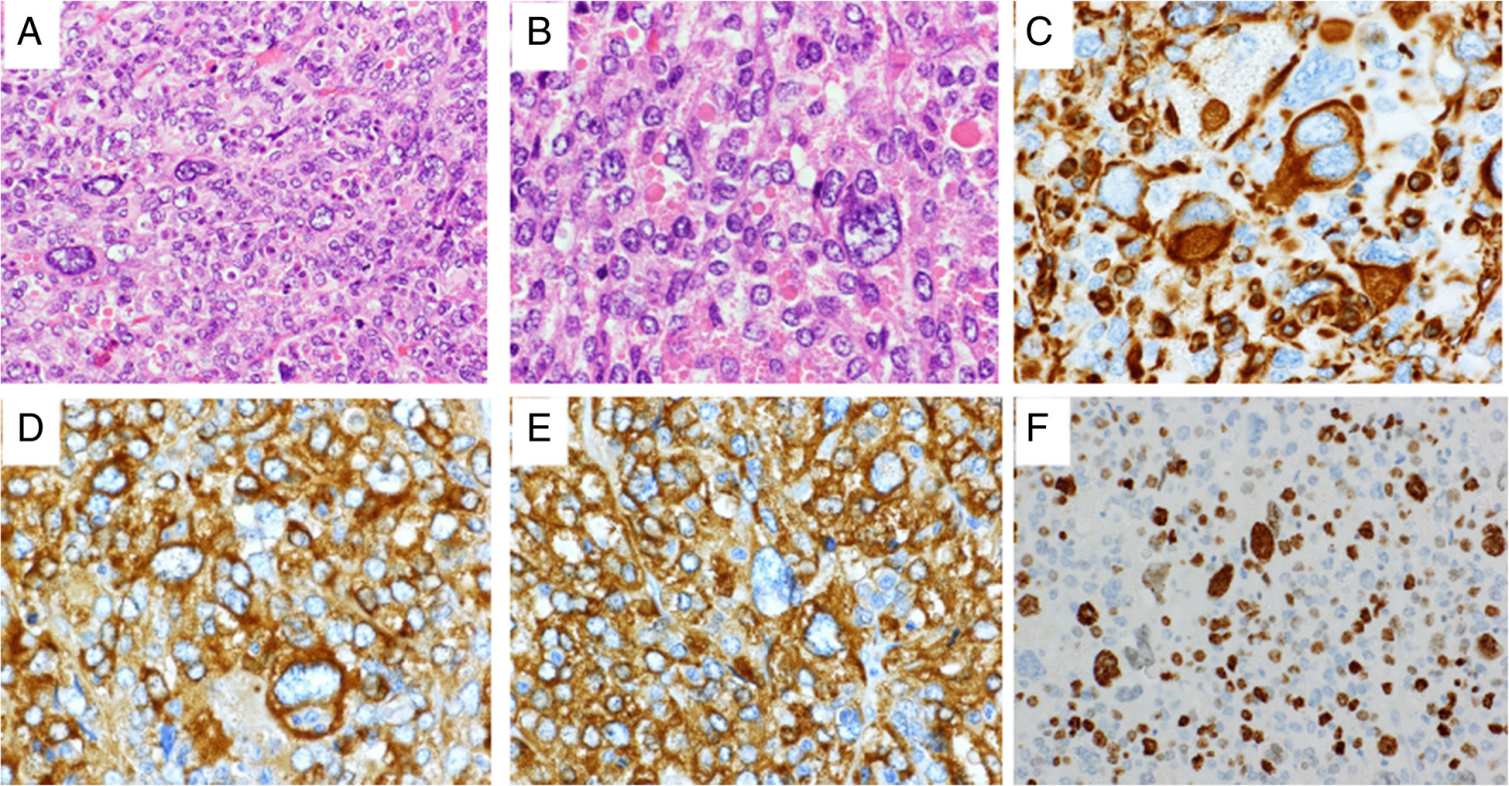
Histological features of HE staining (×200, **a**; ×400, **b**) showed spindled, oval, or stellate tumor cells distributed in myxoid or fibrous stroma. Nuclear pleomorphism and hyperchromasia with frequent multinucleated or bizarre giant cells were apparent. Multiple varying-sized cytoplasmic eosinophilic globules were seen. The tumor cells were positive for vimentin (×400, **c**), alpha 1-antitrypsin (×400, **d**), and alpha 1-antichymotrypsin (×400, **e**). The MIB-1 index was 30 % (×200, **f**)

**Fig. 6:**
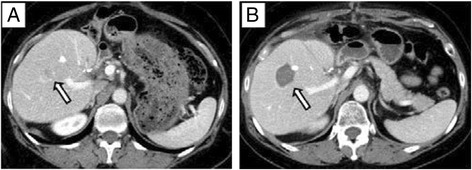
The recurred tumor (*arrow*, **a**) was successfully treated with radio-frequency ablation (*arrow*, **b**).

A 53-year-old female was admitted to our hospital with a huge liver tumor with symptoms of epigastralgia and back pain. Her HBs-Ag and HCV-Ab were negative. Serum level of PIVKA-II was elevated to 10,369 mAU/ml, and AFP, CEA, and CA19-9 were in the normal range. CT showed a huge low-density tumor sized 18 × 14 cm that showed mild enhancement after administration of contrast medium (Fig. 4a). MR imaging demonstrated the heterogeneous tumor to be hypointense on T1-weighted images (Fig. 4b) and hyperintense on T2-weighted images (Fig. 4c). Some satellite lesions suspected as cavernous hemangiomas were identified in the bilateral lobe of the liver. The first extended left hepatectomy was performed. Operation time was 459 min and intraoperative bleeding was 877 g. Resected specimen revealed a tumor with multiple components, sized 22 × 19 × 14 cm (Fig. 4d). Histologically, spindled, oval, or stellate tumor cells were distributed in myxoid or fibrous stroma. Nuclear pleomorphism and hyperchromasia with frequent multinucleated or bizarre giant cells were apparent. Multiple varying-sized cytoplasmic eosinophilic globules were seen (Fig. 5a, b). The tumor cells were positive for vimentin (Fig. 5c), alpha 1-antitrypsin (Fig. 5d), alpha 1-antichymotrypsin (Fig. 5e), and desmin, and partially positive for CD34 and alpha-SMA. The MIB-1 index was 30 % (Fig. 5f). Hepatocyte specific antigen (HSA) and cytokeratin were negative in the tumor. The tumor was histologically diagnosed as a UESL in the normal liver. Three months after the first operation, a TACE (CDDP 80 mg + lipiodol 4 ml, 5-FU 1000 mg, and gelatin sponge) was performed. Four and 14 months after the first operation, a partial hepatic resection of segments 6 and 7 and a partial hepatic resection of segment 8 in combination with RFA (pre, Fig. 6a; post, Fig. 6b) were also performed, respectively. She is now alive without any recurrent diseases for more than 65 months from the initial hepatic resection.

## Conclusions

UESL in adult is extremely rare and the prognosis of UESL patients is very poor [3, 4, 7]. Although the treatment strategy of adult UESL is not well established, if possible, liver resection and adjuvant chemotherapy are recommended [7].

Two cases of adult UESL patients were occasionally admitted to our hospital in the same period. Instead of the huge size of the tumors, both patients were successfully treated with the initial hepatic resections. The first patient survived 62 months with receiving a total six times of hepatic resection, twice RFAs, and two times of TACEs. The second patient received three times of hepatic resection and a TACE and RFA and is still alive more than 65 months from the initial treatment. Both patients received no adjuvant chemotherapy or radiotherapy.

UESL has been reported to be chemotherapy-sensitive or is highly sensitive to TACE [3, 7]. However, the reported regimens of chemotherapy for adult UESL were heterogeneous [7]. As systemic chemotherapy, sarcoma-directed one such as vincristine, actinomycin, ifosfamide, doxorubicin, carboplatin, or etoposide was often selected for metastatic UESL [7]. In the current patients, TACE was selected to treat the intrahepatic multiple recurrences of the UESL. Sarcoma-directed chemo-drugs were not established as the regimens of TACE, so a combination of CDDP, epirubicin, and 5-FU was used [8]. In the current two patients, TACE was not so effective; thereafter, repeated hepatic resections or RFAs were mainly selected.

Noguchi et al. reported a long-term survival case of adult UESL treated with hepatic resection, adjuvant chemotherapy, and additional radiotherapy [9]. They also reviewed the reported long-term survival cases of adult UESL and discussed that complete resection is important for ensuring long-term survival. In the treatment strategy of liver tumors, the effectiveness of RFA as well as surgical resection is widely reviewed [10]. To the best of our knowledge, there are no English reports of adult UESL treated with RFA. In the current cases, four tumors treated with RFA showed no recurrences at the therapeutic sites for mean observation period of 35 months. RFA can be one of the effective modalities to treat UESL, especially in cases with multiple small intrahepatic recurrences. Repeated hepatectomies and RFAs for the recurrent diseases may influence such long survivals of the current adult UESL patients.

In conclusion, although adult UESL is a rare and aggressive malignancy, repeated hepatic resections and RFAs for the recurrent diseases may provide a longer survival.

## Consent

Written informed consents were obtained from patients and their family for publication of this case report and any accompanying images. Copies of the written consents are available for review by the Editor-in-Chief of this journal.

## Abbreviations

AFP: alpha-fetoprotein
CA19-9: carbohydrate antigen 19–9
CEA: carcinoembryonic antigen
CT: computed tomography
HBs-Ag: hepatitis B virus surface antigen
HCV-Ab: hepatitis C virus antibody
MR: magnetic resonance
PIVKA-II: protein induced by vitamin K absence
RFA: radio-frequency ablation
TACE: transarterial chemoembolization
UESL: undifferentiated embryonal sarcoma of the liver
